# Improved accuracy and precision of fat-suppressed isotropic 3D T2 mapping MRI of the knee with dictionary fitting and patch-based denoising

**DOI:** 10.1186/s41747-023-00339-8

**Published:** 2023-05-22

**Authors:** Simon Kuhn, Aurélien Bustin, Aicha Lamri-Senouci, Simone Rumac, Jean-Baptiste Ledoux, Roberto Colotti, Jessica A. M. Bastiaansen, Jérôme Yerly, Julien Favre, Patrick Omoumi, Ruud B. van Heeswijk

**Affiliations:** 1 Department of Radiology, Lausanne University Hospital (CHUV) and University of Lausanne (UNIL), Lausanne, Switzerland; 2 Department of Cardiovascular Imaging, Hôpital Cardiologique du Haut-Lévêque, CHU de Bordeaux, France; 3 IHU LIRYC, Electrophysiology and Heart Modeling Institute, INSERM U1045, Centre de Recherche Cardio-Thoracique de Bordeaux, Université de Bordeaux, Bordeaux, France; 4 Center for BioMedical Imaging (CIBM), Lausanne, Switzerland; 5 Biomedical Data Science Center (BDSC), Lausanne University Hospital (CHUV) and University of Lausanne (UNIL), Lausanne, Switzerland; 6 Department of Diagnostic, Interventional and Pediatric Radiology (DIPR), Bern University Hospital, University of Bern, Inselspital, Switzerland; 7 Translation Imaging Center (TIC), Swiss Institute for Translational and Entrepreneurial Medicine, Bern, Switzerland; 8 Department of Musculoskeletal Medicine, Lausanne University Hospital (CHUV) and University of Lausanne (UNIL), Lausanne, Switzerland; 9 The Sense Innovation and Research Center, Lausanne and Sion, Switzerland

**Keywords:** Cartilage, Knee joint, Magnetic resonance imaging, Phantoms (imaging)

## Abstract

**Purpose:**

To develop an isotropic three-dimensional (3D) T2 mapping technique for the quantitative assessment of the composition of knee cartilage with high accuracy and precision.

**Methods:**

A T2-prepared water-selective isotropic 3D gradient-echo pulse sequence was used to generate four images at 3 T. These were used for three T2 map reconstructions: standard images with an analytical T2 fit (AnT2Fit); standard images with a dictionary-based T2 fit (DictT2Fit); and patch-based-denoised images with a dictionary-based T2 fit (DenDictT2Fit). The accuracy of the three techniques was first optimized in a phantom study against spin-echo imaging, after which knee cartilage T2 values and coefficients of variation (CoV) were assessed in ten subjects in order to establish accuracy and precision *in vivo*. Data given as mean ± standard deviation.

**Results:**

After optimization in the phantom, whole-knee cartilage T2 values of the healthy volunteers were 26.6 ± 1.6 ms (AnT2Fit), 42.8 ± 1.8 ms (DictT2Fit, *p* < 0.001 *versus* AnT2Fit), and 40.4 ± 1.7 ms (DenDictT2Fit, *p* = 0.009 *versus* DictT2Fit). The whole-knee T2 CoV reduced from 51.5% ± 5.6% to 30.5 ± 2.4 and finally to 13.1 ± 1.3%, respectively (*p* < 0.001 between all). The DictT2Fit improved the data reconstruction time: 48.7 ± 11.3 min (AnT2Fit) *versus* 7.3 ± 0.7 min (DictT2Fit, *p* < 0.001). Very small focal lesions were observed in maps generated with DenDictT2Fit.

**Conclusions:**

Improved accuracy and precision for isotropic 3D T2 mapping of knee cartilage were demonstrated by using patch-based image denoising and dictionary-based reconstruction.

**Key points:**

• Dictionary T2 fitting improves the accuracy of three-dimensional (3D) knee T2 mapping.

• Patch-based denoising results in high precision in 3D knee T2 mapping.

• Isotropic 3D knee T2 mapping enables the visualization of small anatomical details.

**Supplementary Information:**

The online version contains supplementary material available at 10.1186/s41747-023-00339-8.

## Background

The prevalence of knee osteoarthritis (OA) has seen a significant increase in modern industrial societies [1]. Common identified risk factors include old age and obesity [2, 3], both of which have seen an upward trend in recent times [4, 5], foreshadowing a further increase in knee OA around the world. Finding appropriate methods for a detection of cartilage alterations at the early stages of the disease is therefore an increasingly relevant issue, since cartilage has a limited ability to regenerate once damaged.

Magnetic resonance imaging (MRI) is the reference standard for the radiation-free compositional analysis of cartilage as well as the whole-knee assessment of OA [6]. Together with T1-rho and delayed-gadolinium-enhancement mapping [7], T2 mapping is one of the most widespread methods for the detection of compositional alteration of cartilage at the early stages of OA [8–10]. Here, damaged cartilage shows longer T2 relaxation times compared to healthy cartilage [11]. To fully assess the complex three-dimensional (3D) structure of cartilage, isotropic 3D T2 mapping sequences have been developed and have the advantage of maintaining the same spatial resolution when reformatting the map in any direction [9]. This is however achieved at the cost of long acquisition times (> 10 min), a relatively poor precision (standard deviations of ~ 20%), as well as moderate accuracy (bias of ~ 20%). In addition, these techniques use a non-linear analytic fit to compute T2 relaxation times [10, 12], which prolongs the map reconstruction time.

The goal of this study was therefore to remove several of the drawbacks associated with current isotropic 3D T2 mapping by improving the technique in two steps. As a first step, a dictionary was generated through numerical simulation of the Bloch equations [13]. This dictionary was then calibrated to improve the T2 mapping accuracy. This technique also has the benefit of decreasing the map reconstruction time. As a second step, the precision was improved by using the high-dimensionality undersampled patch-based reconstruction (HD-PROST) denoising algorithm [14], which compares voxel patches of a selected size across all dimensions to detect similar patterns and separates the noise from the relevant signal. These techniques and their combination were first optimized in phantom studies and then validated on selected volunteers, with and without MRI signs of mild knee OA.

## Methods

### Pulse sequence and image acquisition

**Fig. 1:**
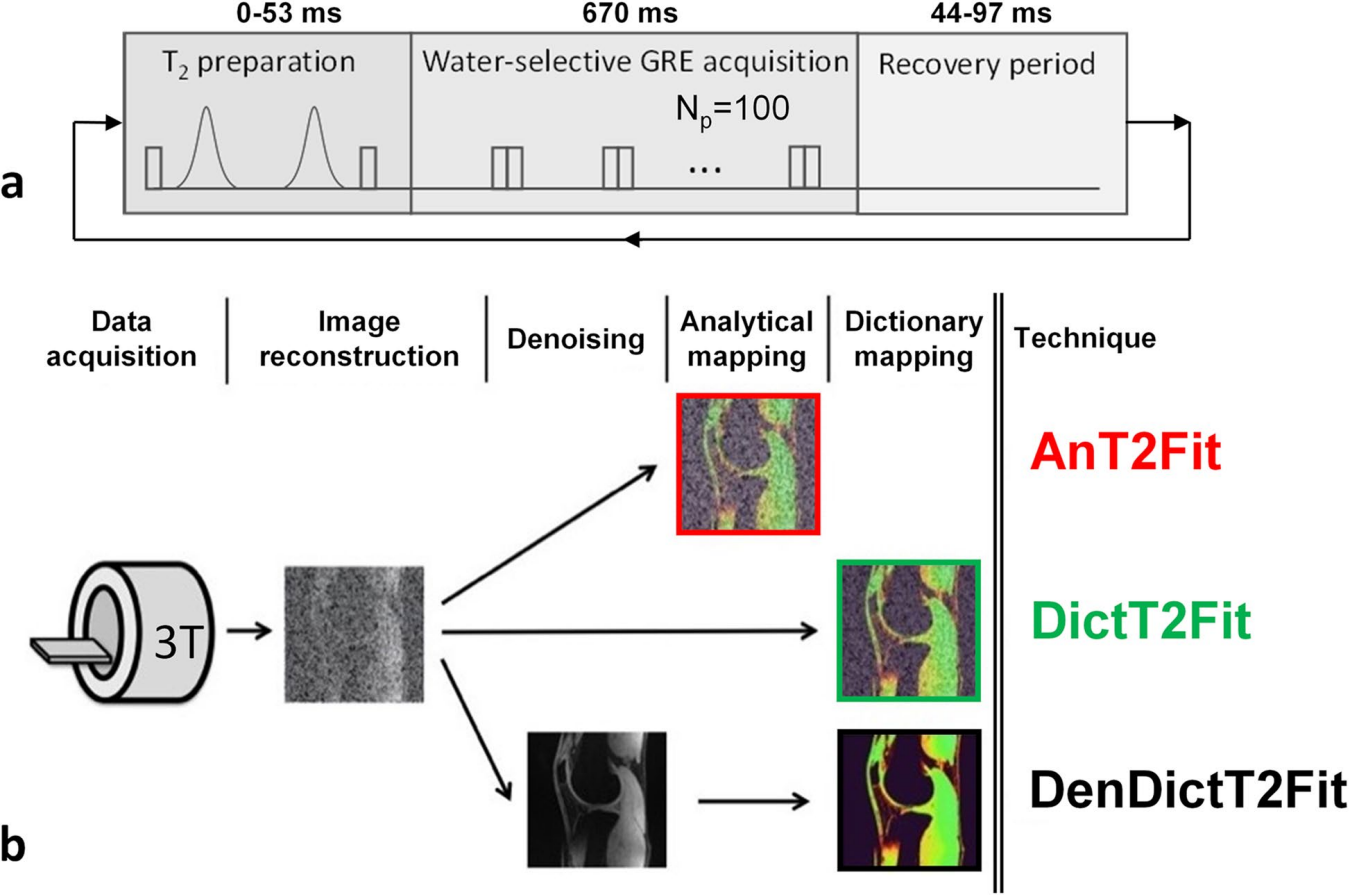
The Lib3DGRE pulse sequence used for T2 mapping of the knee and the following reconstruction techniques. **a** An adiabatic T2-prep module with variable duration precedes a GRE segment of *Np* = 100 readouts with LIBRE water excitation. This is followed by a period to recover magnetization and to lower the effective energy deposition (SAR) of the T2-prep. Note that the total segment duration is fixed. Echo time *TE* = 3.24 ms, *TR* = 6.96 ms, receiver bandwidth 301 Hz/px. **b** The three image reconstruction and mapping techniques compared in this study are as follows: (I) an analytic map of a standard GRAPPA-reconstructed image (AnT2Fit); (II) a dictionary map of the same image (DictT2Fit); and (III) a dictionary map of an HD-PROST-denoised version of that image (DenDictT2Fit). The color coding (red, green, black) of the three techniques is used throughout this study. *GRAPPA* Generalized autocalibrating partially parallel acquisitions, *GRE* Gradient-recalled echo, *HD-PROST* High-dimensionality undersampled patch-based reconstruction, *Lib3DGRE* LIBRE-enabled three-dimensional GRE pulse sequence, *LIBRE* Lipid-insensitive binomial off-resonant radiofrequency excitation, *SAR* Specific absorption rate, *T2-prep* T2 preparation

Data were acquired on a 3-T clinical system (Magnetom PrismaFit, Siemens Healthcare), using a 15-channel transmit-receive knee coil (Quality Electrodynamics). A 0.6 mm^3^ isotropic 3D segmented gradient-recalled echo (GRE) pulse sequence with lipid-insensitive binomial off-resonant radiofrequency excitation (LIBRE) water-selective RF pulses [10, 15] and variable adiabatic T2 preparation (T2-prep) time was used with centric Cartesian *k*-space ordering (Fig. 1a). We named this sequence Lib3DGRE. Further acquisition parameters included *Np* = 100 readouts per segment, *k*_*y*_ GRAPPA (generalized autocalibrating partially parallel acquisitions) [16] factor *R* = 2, four T2-prep times with echo time (*TE*)_*T2-prep*_ = {0, 23, 38, 53} ms, recovery period between GRE segments = 44–97 ms, matrix 272 × 280 × 144, and total scan time 11.1 min.

In the phantom experiments conducted for calibration purposes, two sequences were used as reference standard scans: (i) a two-dimensional spin-echo (SE) T2 mapping pulse sequence with echo time (*TE)* = 10–300 ms in exponential steps and repetition time (*TR*) = 10 s) and (ii) a two-dimensional inversion-recovery turbo spin-echo (TSE) T1 mapping pulse sequence with inversion time = 10–7,000 ms in exponential steps and *TR* = 12 s.

### Image reconstruction

Images were reconstructed via a standard 3D GRAPPA algorithm, using a kernel of size 3 × 2 × 3 in [*k*_*x*_-*k*_*y*_-*k*_*z*_]. These will hereafter be called *standard images*. These images were then processed with the HD-PROST patch-based denoising algorithm [14], resulting in sets of four new denoised images. Briefly, HD-PROST works by extracting similar square patches from the multi-contrast images, which are reformatted as a complex-valued high-order low-rank tensor. This tensor is then compressed through high-order tensor decomposition, resulting in highly effective denoising compared to traditional single-contrast magnitude-image denoisers such as BM3D [17]. The resulting images will hereafter be called *denoised images*. To shorten the reconstruction time, two-dimensional denoising was performed slice-by-slice in the sagittal plane, since the improvements of a full 3D application (*i.e.*, using cubic patches and a volume-wide search) were found to be negligible in prior studies [18].

The denoising parameters for HD-PROST were calibrated using the reconstructed image of a randomly chosen healthy volunteer. The parameters were initiated from previous successful applications [14] and chosen to minimize the standard deviation of the intensity within a uniform region of knee cartilage while visually ensuring the absence of any blurring effects. Denoising parameters [14] included a regularization factor $\lambda =1$, a patch size of 5px × 5px, and a maximum number of 20 similar patches to select. All reconstructions and analyses were performed in MATLAB (The MathWorks, Inc.) on a workstation equipped with two Intel Xeon CPUs, 512 GB of RAM, and an NVIDIA Tesla K40 graphics processing unit.

### T2 map calibration and reconstruction

The previously studied analytic T2 fit with an offset δ to account for T1 recovery [10, 12, 19, 20] was used to generate a T2 map from the four standard images:

1 $$S\left({TE}_{T2prep}\right)=S_0\;\cdot\left(e^{-\frac{TE_{T2prep}}{T_2}}+\frac\delta{T_2}\right),$$

where ${S}_{0}=S\left(T{E}_{T2prep}=0\right)$ and the offset δ was calibrated with the use of a NIST phantom (System Standard Model 130, QalibreMD). For this calibration, images of the NIST phantom were acquired with both the Lib3DGRE and gold-standard SE T2 mapping sequences. We focused on the five compartments within the “T2 layer” of the phantom with SE-determined T2 values closest to those of cartilage in the human knee (20–90 ms).

To calibrate the offset of the analytic fit, a T2 map of a standard image of the phantom was generated for δ = 1, 2…10 ms. The average T2 values in each relevant compartment were then linearly correlated with those from the SE reference map. The value of δ that resulted in a linear fit closest to the identity line was retained for the volunteer studies.

For the second T2 mapping method, numerical simulations of the pulse sequence with the Bloch equations (“Bloch simulations”) were performed to create a dictionary with the T2 relaxation time varying between 5 and 660 ms in increasing steps. This dictionary was matched [21] to the absolute signal evolution in each voxel through the four source images to determine the most likely T2 relaxation time and thus to generate a dictionary-based T2 map.

Since Lib3DGRE uses a centric Cartesian *k*-space trajectory, the *Np* = 100 readouts in a segment should in theory have an exponentially decaying contribution to the signal strength in the dictionary. In most dictionary generation for segmented centric Cartesian readouts, only the first readout is taken into account because the signal amplitude contribution of the rest of the readout train can be neglected. However, with *Np* = 100 readouts this simplification is no longer valid. To address this simplification, and since the T1 recovery and T2* decay as well as their interplay with the phase encoding gradients were not known, the decreasing contribution of the readouts was modeled as an average of the first *N*_*c*_ readouts of the segment. This number of averaged readouts *N*_*c*_ was empirically calibrated by generating a dictionary for *N*_*c*_ = 1, 2…20 and performing a linear fit against the abovementioned SE map in the NIST phantom.

This was performed as follows: For each of the five relevant compartments within the phantom, a series of dictionaries of the Lib3DGRE sequence were generated with *N*_*c*_ = 1:1:20, with the T1 value each time set to the reference value of the considered compartment. The rest of the readouts in the segments was ignored, as they would encode the *k*-space periphery and thus not meaningfully contribute to the signal strength. Linear fits between the SE and dictionary-based T2 values in the phantom compartments were calculated for all *N*_*c*_ values. The dictionary with the *N*_*c*_ value that resulted in the linear fit closest to the identity line was retained for the volunteer studies and was used to map T2 values from both the standard and denoised images.

These calibrations resulted in three mapping techniques that were used in the *in vivo* studies (Fig. 1b): (i) analytic T2 maps of standard images (AnT2Fit); (ii) dictionary-based T2 maps of standard images (DictT2Fit); and (iii) dictionary-based T2 maps of denoised images (DenDictT2Fit). During the application of each of these techniques on *in vivo* data, their reconstruction, denoising, and mapping times were recorded.

### *In vivo* studies

All studies were approved by the local ethics committee (CER-VD, authorization 2019–00291), and all study participants provided written informed consent. A group of 10 subjects (5 female, age 44 ± 14 years) was randomly selected out of a large set of knee scans obtained in a cohort study on asymptomatic volunteers and was estimated to be large enough to show statistically significant differences between the tested techniques. The subjects had no OA (*n* = 5) or mild OA (*n* = 5) at routine MRI, as defined by isolated, focal cartilage lesions on fat-suppressed proton-density sequences acquired in all three planes. None of the subjects showed any other signs of OA such as meniscal or subchondral bone lesions. Regions of interest defining the femorotibial articular cartilage were drawn on the 23-ms T2-weighted source images as a consensus between two radiologists with 1 and 12 years of experience (A.L.S. and P.O. respectively) and a physicist with 1 year of experience (S.K.). These regions of interest were split into lateral and medial femorotibial cartilage, as well as tibial and femoral cartilage, the latter of which was in turn split into anterior, central, and posterior regions, resulting in a total of eight separate regions [22].

T2 maps of knees were computed using the three techniques described above. Dictionary T2 maps were obtained by setting the dictionary T1 relaxation time to its referenced value for healthy cartilage (T1 = 1'098 ms) [23], while the T2 relaxation time was allowed to vary with different step sizes (T2 = 5:1:99, 100:20:300, 360:60:660 ms), performing voxel-wise dictionary matching as described above. For each technique and each subject, the average T2 values within the eight aforementioned regions as well as the whole-knee cartilage were computed as an accuracy indicator. The regional standard deviations were used to calculate the corresponding coefficient of variation (CoV = 100 × standard deviation/average), an inverse indicator of the precision of the technique. Whole-knee T2 values and their CoVs were compared between the different techniques over all subjects.

Map sharpness was assessed for all three techniques by fitting the same vertical line from the middle of the femur to the tibia with a parametrized sigmoid function:

2 $$T_2(x)=\frac a{1+e^{-k\left(b+x\right)}+c},$$

where *a*, *b*, and *c* are scaling variables and *k* is the sharpness (in px^−1^; higher is better) [24].

### Statistics

Results are reported as mean and their standard deviation. Comparisons of two groups were made using one- or two-tailed Student *t*-tests as appropriate, where *p* < 0.05 was considered significant. Multigroup comparisons were made using one- or two-way analysis of variance (ANOVA) as appropriate. The goodness of the fit *R*^*2*^ was calculated for linear regressions.

## Results

### Phantom calibration

**Fig. 2:**
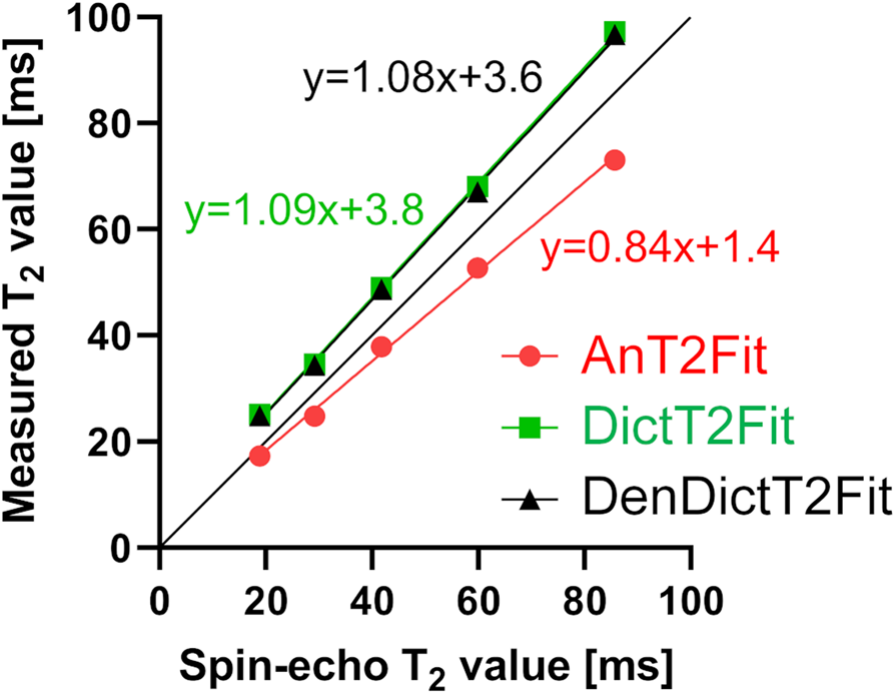
Correlations illustrate the agreement of the optimized T2 mapping methods with the SE reference in the NIST phantom. Both dictionary maps (on standard and denoised phantom images) have a slope closer to the identity line than the analytic map on the standard phantom image, although they come with a slightly larger offset. *R*^2^ > 0.99 for all three fits. *NIST* National Institute of Standards and Technology, *SE* Spin-echo. For the differences between the three techniques, see the legend of Fig. 1

The optimal offset $\delta$ in Eq. 1 for the analytical fitting method was found to be $\delta$ = 5 ms. While closest to the identity line of the possible fits in the phantom, the analytic T2 mapping method slightly underestimates the SE T2 values and blunts the response with a slope of 0.84 (Fig. 2). For the dictionary-based T2 maps, the number of averaged *k*-space lines *N*_*c*_ = 5 resulted in the fit that was most similar to the identity line with slopes of 1.09, resulting in a slight overestimation of the SE-based T2 values (Supplementary Table S1). Finally, the denoising slightly improved the fit with a slope of 1.08.

### *In vivo* studies

**Fig. 3:**
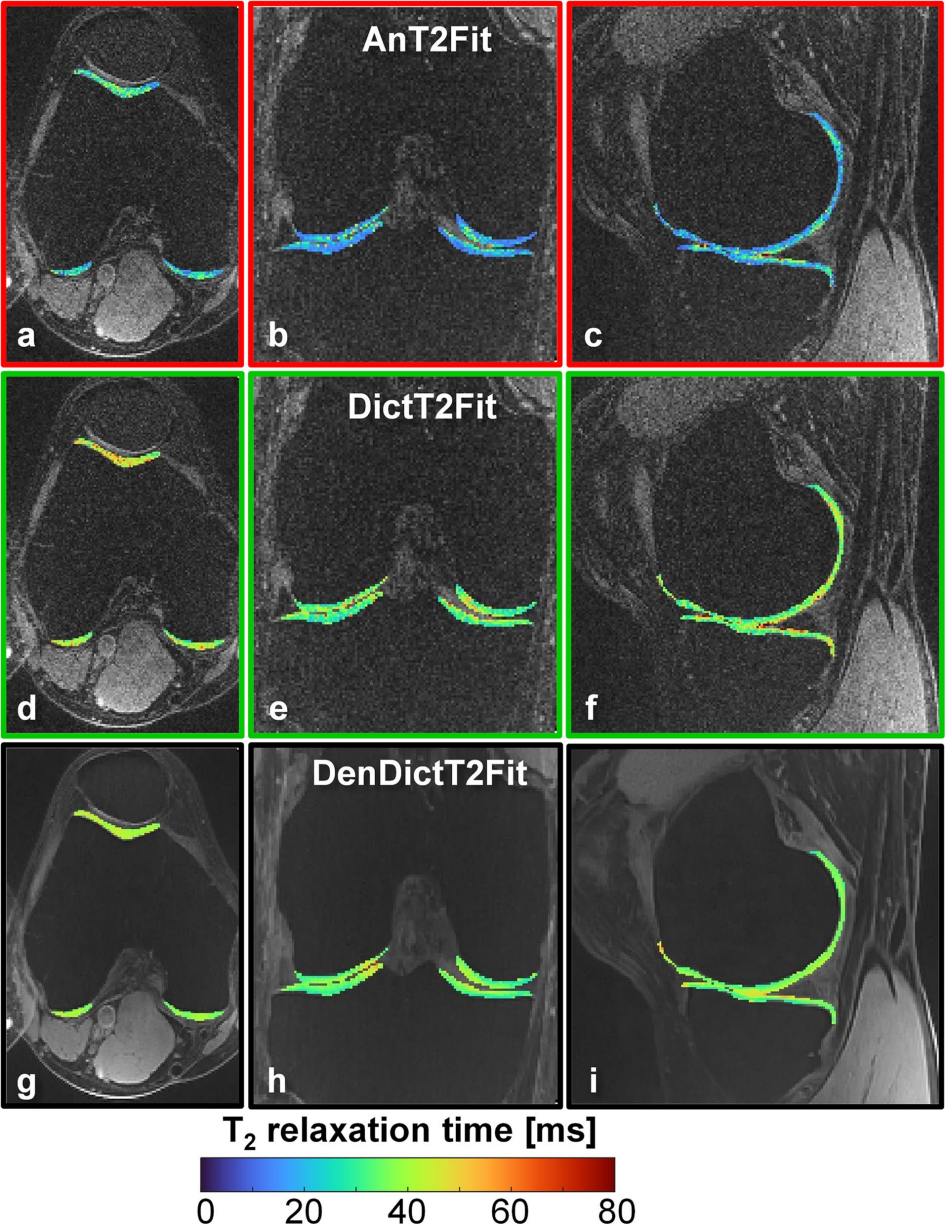
Orthogonal T2 maps of the knee of a volunteer without osteoarthritis lesions, superimposed on top of fat-suppressed T2-weighted source images. **a**–**c** Analytic maps based on standard images (AnT2Fit). **d**–**f** Dictionary-based maps of the same standard images (DictT2Fit). The T2 values are visibly more homogeneous in the different segments, while the average T2 value increased. **g**–**i** Dictionary-based maps of HD-PROST-denoised images (DenDictT2Fit). While the T2 values remain similar, the denoising is also visually apparent. *HD-PROST* High-dimensionality undersampled patch-based reconstruction

**Fig. 4:**
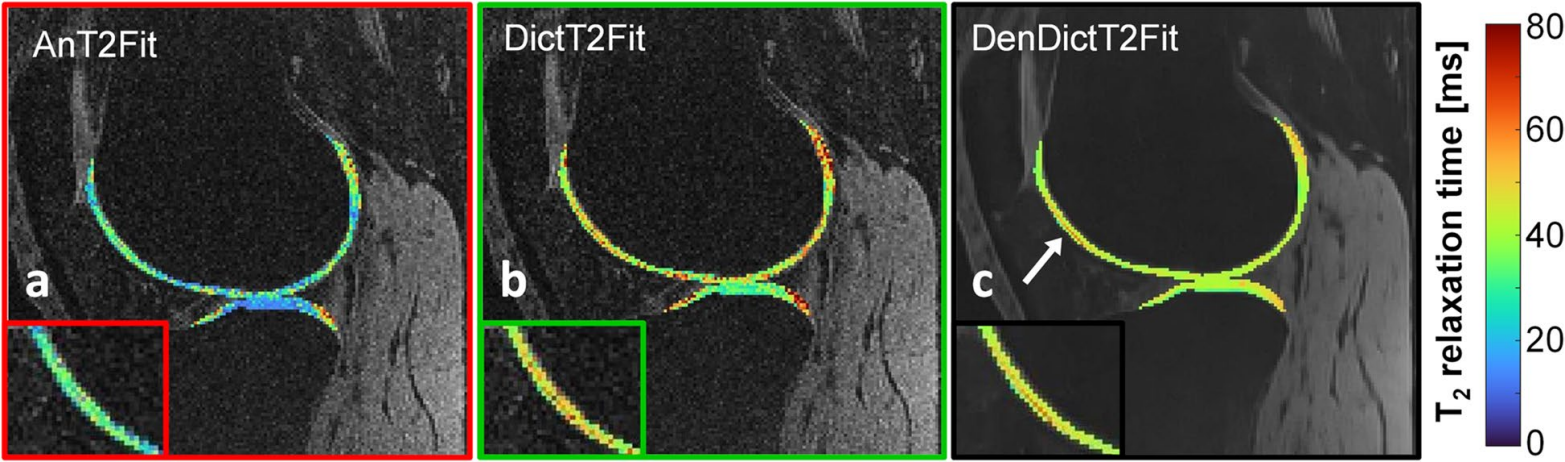
T2 maps of the knee cartilage with a cartilage lesion. **a, b** The maps are superimposed on top of fat-suppressed T2-weighted images that were used to compute the maps. **c** The lower standard deviation of the denoised map reveals an inhomogeneity in the T2 values of the anterior lateral cartilage (white arrow), which was confirmed by a radiologist to be a lesion after reading clinical routine images. The sharp edges of the inhomogeneity also illustrate that the denoising retained the sharpness of the map

**Fig. 5:**
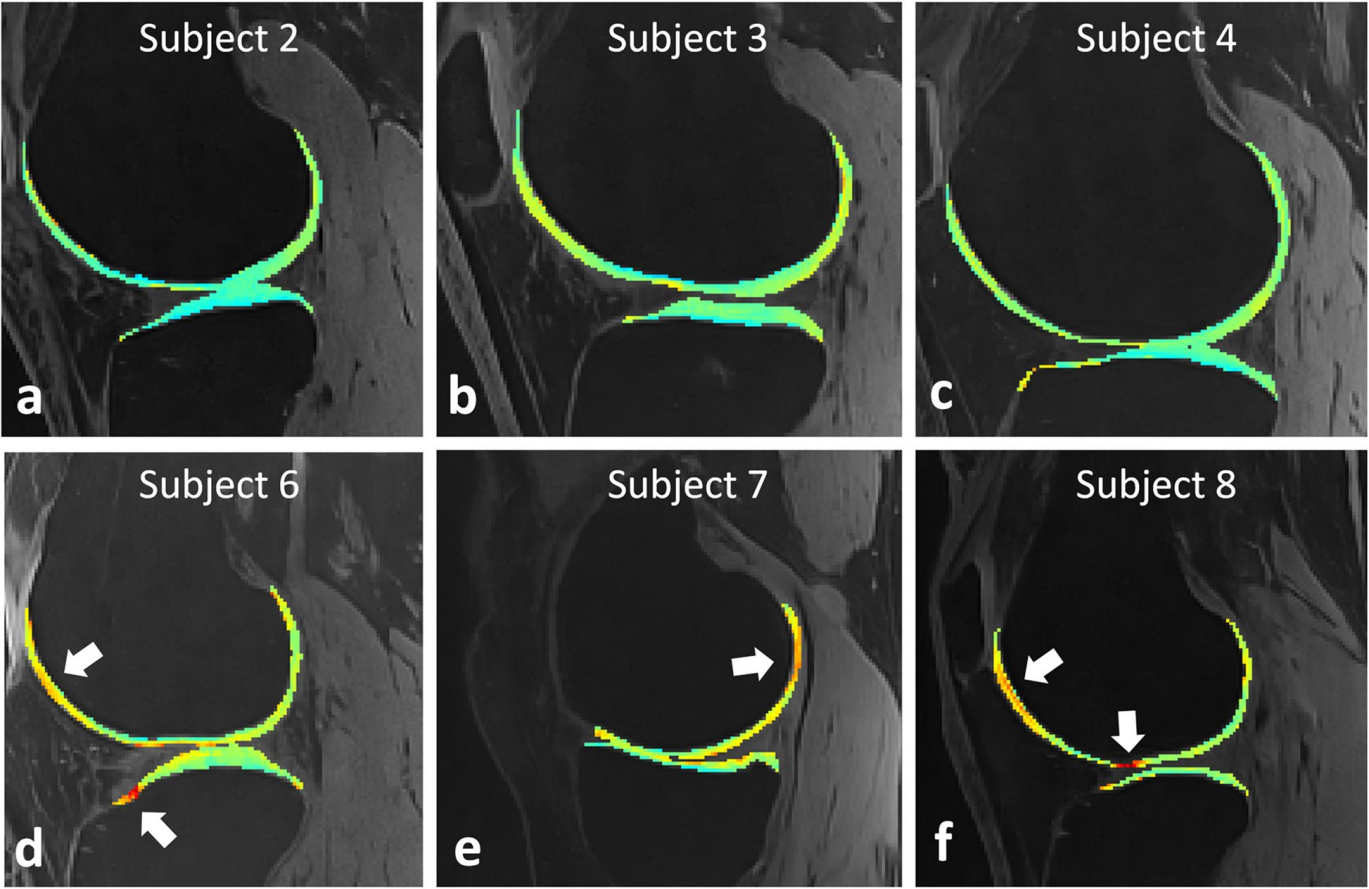
Dictionary-based denoised isotropic T2 maps in six different subjects**.** Robust fat suppression enables the unambiguous delineation of the cartilage, while the dictionary mapping of the denoised images results in smooth maps. **a–c** Representative sagittal reconstructed T2 maps in three healthy subjects in which no focal lesions were detected. The cartilage has smooth and uniform T2 values. **d–f** Sample sagittal reconstructed T2 maps in three subjects with mild osteoarthritis in which lesions were detected (arrows)

The two image reconstructions (standard and denoised) led to visually similarly sharp images in all subjects, while both T2 mapping methods (analytic and dictionary-based) led to visually similarly sharp maps. As expected, the proposed denoised images and dictionary fitting led to smooth cartilage (Fig. 3). Small cartilage lesions that were not visible in the standard maps were observed in the dictionary-based denoised maps in the five mild-OA subjects (Figs. 4 and 5).

**Fig. 6:**
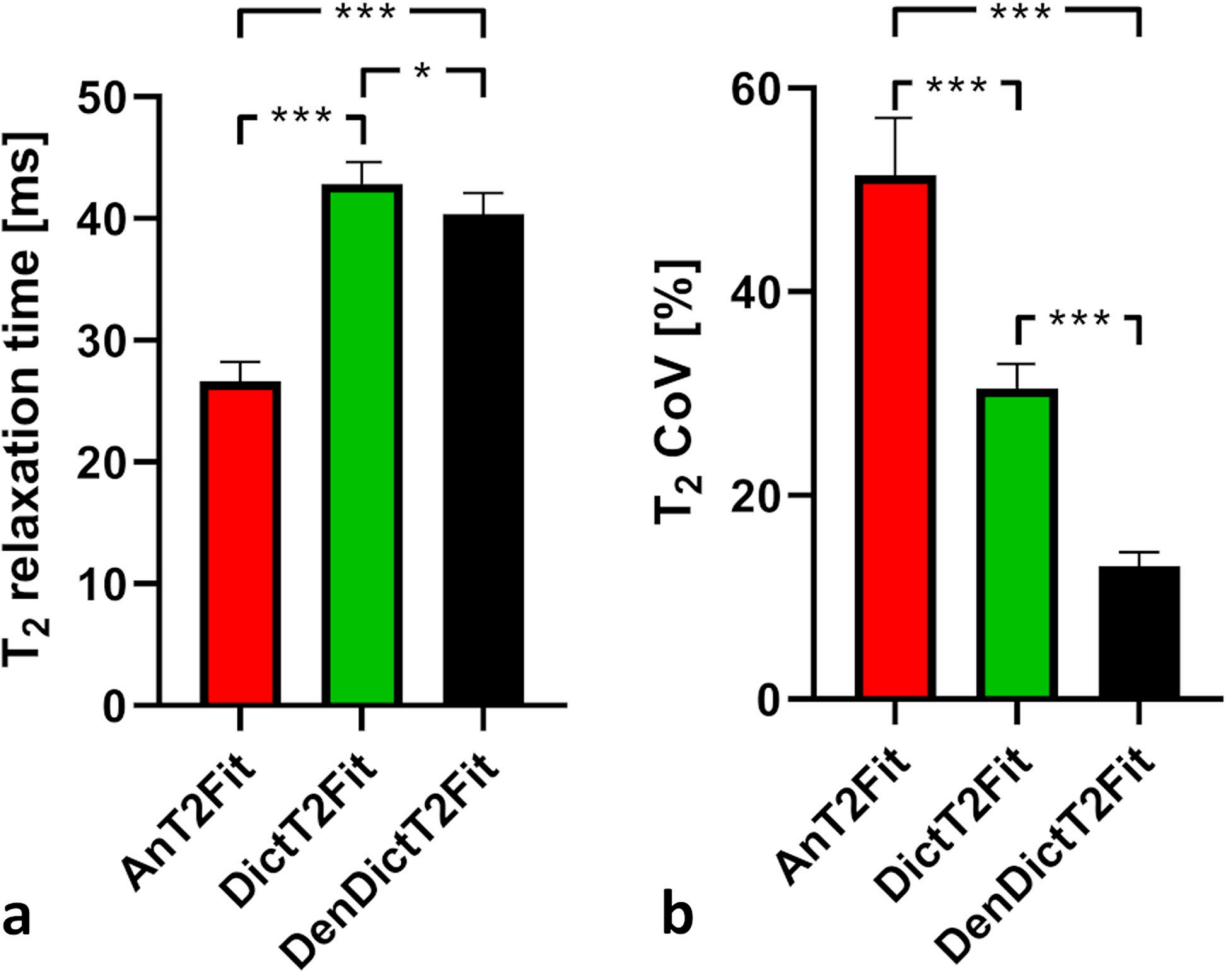
Comparison of the three map reconstruction techniques for knee cartilage T2 relaxation times. **a** Whole-knee results averaged over the ten subjects. The T2 change from one map reconstruction technique to the next was always significant. **b** The precision (assessed as the inverse of the coefficient of variation similarly improves from one technique to the next. *p < 0.05, ***p < 0.001

The whole-knee cartilage T2 values of the three techniques averaged over the ten subjects were 26.6 ± 1.6 ms, 42.8 ± 1.8 ms (*p* < 0.001 *versus* AnT2Fit), and 40.4 ± 1.7 ms (*p* = 0.011 *versus* DictT2Fit), respectively (Fig. 6). The T2 CoV reduced from 51.5 ± 5.6% (AnT2 - Fit) to 30.5 ± 2.4% (DictT2Fit) and finally to 13.1 ± 1.3% (DenDictT2Fit, *p* < 0.001 between all). Similar trends were observed for the different regions of the cartilage (Supplementary Fig. S1). The sharpness measured in the three techniques was *k* = 2.8 ± 0.9 px^−1^ for AnT2Fit, 3.0 ± 0.4 px^−1^ for DictT2Fit, and 3.1 ± 0.4 px^−1^ for DenDictT2Fit (*p* > 0.258 between all three techniques).

The time taken for each step of the data treatment and mapping procedure was logged for all subjects. While the denoising algorithm added time (3.0 ± 0.2 min), it took only half that of the GRAPPA image reconstruction (6.1 ± 0.6 min). The dictionary fit decreased the total mapping time by 97% from 42.6 ± 10.7 min to 1.2 ± 0.1 min. The total map reconstruction times thus decreased significantly from AnT2Fit (48.7 ± 11.3 min) to DictT2Fit (7.3 ± 0.7 min, *p* < 0.001) and increased again for DenDictT2Fit (10.3 ± 0.9 min, *p* < 0.001, Supplementary Table S2).

## Discussion

The aim of this study was to implement and demonstrate accuracy and precision improvements for fat-suppressed 3D isotropic T2 mapping of knee cartilage. The proposed improvements over the reference mapping technique (AnT2Fit) were successful and resulted in more accurate and quickly calculated maps when using dictionary fitting (DictT2Fit), as well as more precise maps when using HD-PROST denoising (DenDictT2Fit).

The phantom experiments demonstrated that all three proposed techniques are sensitive to T2 changes, while the dictionary fits are slightly different from the analytical fit. Furthermore, using the denoised instead of the standard images for the dictionary-based T2 fit did not significantly alter the estimated T2 relaxation time. Despite the optimization of the extra variables in both map reconstructions (δ and *N*_*c*_), neither the analytical fit nor the numerical simulations resulted in a (near-)perfect linear match to the SE technique, and both techniques have a residual bias. This is most likely due to the relatively simple fitting model corrections that these added variables make: δ accounts for the interplay of both non-linear T1 recovery and the effects of the noise floor on the T2 decay [19], while *N*_*c*_ accounts for the magnetization dephasing caused by the encoding gradients. Neither technique takes unknown variables such as magnetization transfer effects or T1 rho relaxation during the adiabatic pulses in the T2 preparation into account. However, the resulting bias of all three techniques is on the same order as the standard deviation of the most precise technique (DenDictT2Fit), indicating that the accuracy is high for all three techniques.

In the *in vivo* studies, the cartilage T2 values obtained with AnT2Fit agreed well with those obtained with T2-prepared GRE in previous studies [9, 10], although they were much lower than what is typically observed with TSE-based techniques [25, 26]. It should be noted that reference-standard SE T2 values cannot be obtained *in vivo* since they typically take several hours to acquire. The T2 values obtained with the dictionary fits (DictT2Fit and DenDictT2Fit) are closer to TSE-based T2 values than the analytical fit. These dictionary-based T2 values were significantly higher than their AnT2Fit counterparts, which we interpret as an improved T2 accuracy. The dictionary-based T2 values were still lower than those obtained with TSE-based techniques, which can be explained with the contributions of uncancelled stimulated echoes to the latter, which slightly increases TSE-based T2 values [9].

Individual lesions were readily visualized as elevated T2 relaxation times with the DenDictT2Fit technique even at a thickness of a few pixels, which reinforces the assertion that the patch-based denoising does not cause significant blurring. The *in vivo* T2 CoV decreased from AnT2Fit to DictT2Fit to DenDictT2Fit, demonstrating the expected improved precision.

This study also has several limitations. While we included asymptomatic subjects both without and with mild OA, the small number of subjects (five of each type) would not allow for the discrimination between these groups, which would be of clinical interest. It is to be emphasized however that all subjects were asymptomatic and the observed isolated cartilage lesions likely represent very early stages of OA. Furthermore, we claim increased accuracy *in vivo* through comparison to literature values, and no direct comparison to an *in vivo* reference standard was made. Finally, the dictionary fit for *in vivo* subjects assumes that the knee cartilage has the same fixed T1 value in all subjects and regions. An optimally accurate application of the dictionary-based mapping method would most likely require specific knowledge of the T1 values within each cartilage region of each subject. To achieve this, the dictionary match could be expanded to also fit the T1 relaxation time, although this added degree of freedom would likely cost precision. Furthermore, an inversion or saturation module would potentially need to be added to create sufficient T1-dependent contrast, which might also lower precision.

The HD-PROST algorithm was highly successful in its patch-based denoising in this novel setting of knee mapping, likely due to the lack of motion and the large image size in which the patch search could be performed. Its regularization parameters were manually and visually optimized, and an automated approach might result in even better performance in future studies. The use of a time-efficient two-dimensional version of the algorithm did not result in any visual artifacts in or near cartilage on the images or maps.

Nevertheless, further improvements in the T2 precision might be obtained by applying a full 3D denoising at the cost of longer image reconstruction times. The resulting high precision also suggests that compressed sensing and the associated scan time reduction could be investigated. The total scan time of over 10 min is prohibitive for most routine clinical protocols, and shortening the acquisition time to below 5 min might result in a more widespread adoption. Especially the use of different semi-random undersampling patterns for the four T2-prepared volumes [27] would allow for the optimization of the scan time. However, it should be noted that the high isotropic spatial resolution would also allow the source images or even the map to be direct replacement for T2-weighted anatomical images.

In conclusion, we demonstrated improved accuracy and precision for isotropic 3D T2 mapping of knee cartilage by using patch-based image denoising and dictionary-based reconstruction.

## Supplementary Information

**Additional file 1: Supplementary Table 1.** Average T1 and T2 values for each of the five compartments in the NIST phantom, according to the methods used to obtain the maps. **Supplementary Table 2.** The computational time taken by each step of the three techniques to produce T2 maps of a single subject. **Supplementary Fig. 1.** A regional comparison of the three T2 mapping techniques in knee cartilage.

## Data Availability

The datasets used and analyzed during the current study are available from the corresponding author on reasonable request.

## Abbreviations

3D: Three-dimensional
AnT2Fit: Analytical T2 map of standard images
CoV: Coefficient of variation
DenDictT2Fit: Dictionary-based T2 map of denoised images
DictT2Fit: Dictionary-based T2 map of standard images
GRAPPA: Generalized autocalibrating partially parallel acquisitions
GRE: Gradient-recalled echo
HD-PROST: High-dimensionality undersampled patch-based reconstruction
Lib3DGRE: LIBRE-enabled 3D GRE pulse sequence
LIBRE: Lipid-insensitive binomial off-resonant radiofrequency excitation
MRI: Magnetic resonance imaging
OA: Osteoarthritis
SE: Spin-echo
T2-prep: T2 preparation
TSE: Turbo spin-echo
