# Supplementary material for: Improved accuracy and precision of fat-suppressed isotropic 3D T2 mapping MRI of the knee with dictionary fitting and patch-based denoising
Source: Eur Radiol Exp. 2023 May 22;7:25. doi: 10.1186/s41747-023-00339-8 (PMC10200730; doi:10.1186/s41747-023-00339-8)
Supplement: Supplementary file 1 — Additional file 1: Supplementary Table 1. Average T1 and T2 values for each of the five compartments in the NIST phantom, according to the methods used to obtain the maps. Supplementary Table 2. The computational time taken by each step of the three techniques to produce T2 maps of a single subject. Supplementary Fig. 1. A regional comparison of the three T2 mapping techniques in knee cartilage. [file 41747_2023_339_MOESM1_ESM.pdf]

ELECTRONIC SUPPLEMENTARY MATERIAL

Improved accuracy and precision in fat-suppressed isotropic 3D T_2 mapping MRI of the knee with dictionary fitting and patch-based denoising

Supplementary Table 1. Average T_1 and T_2 values for each of the five compartments in the NIST phantom, according to the methods used to obtain the maps.

The results also depend on whether the mapped image is noisy or denoised. IR-TSE = inversion-recovery turbo spin echo, NIST = National Institute of Standards and Technology, SE = spin echo.

	**Compartments**				
	**1**	**2**	**3**	**4**	**5**
**T_1 from IR-TSE [ms]**	1025.3	789.1	601.6	448.4	307.8
**T_2 from SE [ms]**	85.7	59.9	41.8	29.2	18.9
**T_2 from technique AnT2Fit [ms]**	73.0	52.7	37.9	24.8	17.3
**T_2 from technique DictT2Fit [ms]**	97.2	68.0	49.0	34.6	25.1
**T_2 from technique DenDictT2Fit [ms]**	96.7	67.0	48.7	34.4	24.9

Supplementary Table 2. The computational time taken by each step of the three techniques to produce T_2 maps of a single subject.

The dictionary mapping time does not include the time taken for the generation of the dictionary itself, which was only done once, since the same dictionary is used for all in vivo maps. The variability is most likely caused by other ongoing computing tasks.

	**AnT2Fit**	**DictT2Fit**	**DenDictT2Fit**
**Image reconstruction [min]**	6.1 ± 0.6	6.1 ± 0.6	6.1 ± 0.6
**Image denoising [min]**			3.0 ± 0.2
**Mapping [min]**	42.6 ± 10.7	1.2 ± 0.1	1.2 ± 0.1
**Total time [min]**	48.7 ± 11.3	7.3 ± 0.7	10.3 ± 0.9

Supplementary Figure 1. A regional comparison of the three T_2 mapping techniques in knee cartilage.

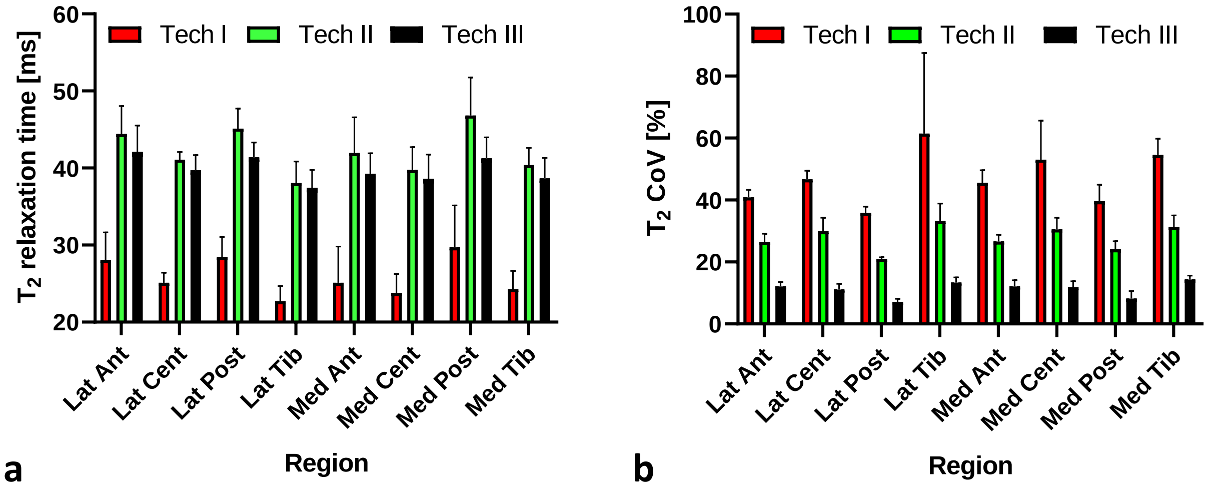

Knee cartilage was divided into eight regions: first into lateral (Lat) and medial (Med), then further into anterior femoral (Ant), central femoral (Cent), posterior femoral (Post) and tibial (Tib).

a. The difference in T_2 values between AnT2Fit (Tech I) and DictT2Fit (Tech II) was significant for every region (all P<0.001). However, the difference between DictT2Fit (Tech II) and DenDictT2Fit (Tech III) was only significant for Lat Post, Med Ant, and Med Post (P<0.036 for these 3 regions).

b. The differences between CoV values were all significant (all P<0.002).
